# Adaptive recognition of machining features in sheet metal parts based on a graph class-incremental learning strategy

**DOI:** 10.1038/s41598-024-61443-2

**Published:** 2024-05-09

**Authors:** Liuhuan Ma, Jiong Yang

**Affiliations:** Zhengzhou University, School of Mechanical and Power Engineering, Zhengzhou, 450001 China

**Keywords:** CAPP, Deep learning, AFR, Graph neural network, Incremental learning, Mechanical engineering, Software

## Abstract

The integration of computer-aided design (CAD), computer-aided process planning (CAPP), and computer-aided manufacturing (CAM) systems is significantly enhanced by employing deep learning-based automatic feature recognition (AFR) methods. These methods outperform traditional, rule-based approaches, particularly in handling the complexities of intersecting features. However, existing deep learning-based AFR methods face two major challenges. The initial challenge stems from the frequent utilization of voxelized or point-cloud representations of CAD models, resulting in the unfortunate loss of valuable geometric and topological information inherent in original Boundary representation (B-Rep) models. The second challenge involves the limitation of supervised deep learning methods in identifying machining features that are not present in the predefined dataset. This constraint renders them suboptimal for the continually evolving datasets of real industrial scenarios. To address the first challenge, this study introduces a graph-structured language, Multidimensional Attributed Face-Edge Graph (maFEG), crafted to encapsulate the intricate geometric and topological details of CAD models. Furthermore, a graph neural network, Sheet-metalNet, is proposed for the efficient learning and interpretation of maFEGs. To tackle the second challenge, a three-component incremental learning strategy is proposed: an initial phase of pre-training and fine-tuning, a prototype sampling-based replay, and a stage employing knowledge distillation for parameter regularization. The effectiveness of Sheet-metalNet and its complementary incremental learning strategy is evaluated using the open-source MFCAD++ dataset and the newly created SMCAD dataset. Experimental results show that Sheet-metalNet surpasses state-of-the-art AFR methods in machining feature recognition accuracy. Moreover, Sheet-metalNet demonstrates adaptability to dynamic dataset changes, maintaining high performance when encountering newly introduced features, thanks to its innovative incremental learning strategy.

## Introduction

Computer-aided process planning (CAPP) serves as a bridge between computer-aided design (CAD) and computer-aided manufacturing (CAM), efficiently transforming a design into a rational manufacturing process sequence. It guides the manufacturing process, ultimately resulting in physical components^1^. In comparison to traditional process planning, CAPP is strategically designed to alleviate the burden on manufacturing professionals by automating complex and time-intensive tasks. These tasks encompass blank design, selection of manufacturing techniques, process orchestration, process route formulation, and temporal projections^2^. The adoption of CAPP yields multifaceted benefits, chief among them substantial financial efficiencies, compression of production timelines, and an augmented competitive edge for manufacturing entities.

Bridging the gap between the CAD system (upstream) and with CAM system (downstream), the primary task of the CAPP system is to interpret the CAD model from a manufacturing perspective. However, the original CAD models are typically limited to fundamental geometric information (e.g., points, edges, and surfaces), lacking the advanced manufacturing semantics necessary for production processes. “Interpreting CAD models from a manufacturing perspective” requires the CAPP system to identify machining features embedded within the raw CAD models. These features are characterized by uniform manufacturing methods within continuous regions on mechanical components, such as holes, slots, and chamfers^2^. For manufacturing experts, this transformation is straightforward and intuitive, but for computers, it poses a challenge. Automatic feature recognition (AFR) technology aims to solve this conversion problem.

AFR technology possesses the ability to convert low-level geometric entities from CAD models into machining features. Its primary focus lies in encoding CAD entities into a computational language, inherently comprehensible to digital systems. The challenge lies in enabling computers to interpret this language from a manufacturing perspective for the CAPP system. The current mainstream AFR technologies are divided into two categories: rule-based methods (e.g., syntactic pattern recognition, hint-based, and graph-based) and learning-based methods (e.g., artificial neural networks (ANNs) and convolutional neural networks (CNNs)). Rule-based methods often have a narrow application range, limited to specific features of specific parts, and most of them cannot handle complex machining features such as intersecting features. In contrast, learning-based methods, especially deep learning methods, are more promising because they are not limited to the scope of parts and can handle complex machining features. However, learning-based methods also have the following limitations:

(i) Many deep learning methods to voxelize or convert Boundary Representation (B-Rep) models into point clouds, which is not only computationally intensive and time-consuming but also struggles with processing intersecting machining features. Furthermore, this conversion results in the loss of valuable position, geometry, and topology information originally present in B-Rep models, information crucial in subsequent part machining.

(ii) Deep learning methods usually learn knowledge from large-scale datasets with machining feature labels, which are rarely available in industrial applications. Even if such datasets exist, they are usually small-scale and necessitate continual supplementation with CAD models. Additionally, deep learning methods theoretically face limitations in recognizing machining features absent in the existing dataset. Therefore, whenever encountering new machining features, the data needs to be reorganized and the neural networks retrained from scratch, increasing both time and space costs.

Given the aforementioned issues, this study made the following contributions:

(i) Referring to attribute adjacency graph (AAG) in graph-based feature recognition algorithms, a Multidimensional Attributed Face-Edge Graph (maFEG) was proposed as a graph-based language for B-Rep models. In contrast to voxel and point cloud representations, maFEG preserves completely the geometric and topological information of B-Rep models. Furthermore, graph neural networks (GNNs), a type of neural network designed for graph data, were used to learn the possible patterns of machining features in maFEG. Based on one of the currently most powerful GNNs, graph isomorphism network (GIN)^3^, a GNN specifically for machining feature recognition, Sheet-metalNet, was proposed. This network demonstrates exceptional accuracy in predicting the machining feature category for any face within a B-Rep model.

(ii) Proposing an incremental learning strategy. Assuming the existence of a real industrial parts CAD model dataset with a continuously expanding sample capacity, with the possibility of adding new machining feature samples each time. Through this incremental learning strategy, Sheet-metalNet can directly utilize the parameters of the original network model, a small subset of the original data, and the entirety of the new data, enabling recognition of both new and old machining features, thereby expanding the scope of recognition of the neural network model.

(iii) Additionally, existing deep learning-based feature recognition research had primarily focused on machine parts for metalworking, with scarce attention given to sheet metal parts, another widely used mechanical component in the industrial sector. Therefore, a sheet metal CAD model dataset called SMCAD dataset was created to fill the gap in deep learning-based machining feature recognition research for sheet metal parts.

The rest of this paper is organized as follows: Section "Related work" reviews related work that motivates the proposed methods. Section "Methodology" describes the details of maFEG, Sheet-metalNet, and the incremental learning strategy. Section "Dataset creation" provides an overview and creation steps of the SMCAD dataset. Section "Experimental results and discussion" illustrates the effectiveness of Sheet-metalNet and the incremental learning strategy through experiments on MFCAD++ and SMCAD datasets, accompanied by explanations of the experimental results. Finally, Section "Conclusion" concludes this work and offers insights into potential future directions.

## Related work

### Rule-based AFR methods

Early AFR methods explicitly performed operations according to various logical rules. Their general approach involved defining machining features based on existing experience and knowledge. They proposed certain definitions to define machining features and build corresponding knowledge bases. The target part was converted into a specific representation by this definition and then matched with the predefined machining feature patterns in the knowledge base. Different rule-based AFR methods adopted different definitions. For example: in syntactic pattern recognition methods^4–6^, descriptive semantic primitives and a series of grammar rules were used to define parts and machining features; in graph-based methods^7–9^, B-Rep models of parts and machining features were converted into attribute adjacency graphs (AAGs) and attribute adjacency subgraphs, respectively; in hint-based methods^10–12^, machining features were summarized as hints (geometric or topological information that can prove the existence of this machining feature), heuristic rules were used to generate hints and infer machining feature types based on hints; in volume decomposition methods^13–16^, part models were decomposed into a series of variational convex bodies or unit cells with basic forms according to their volumes. The decomposed volumes were recombined according to predefined rules to construct the machining features of the parts.

Numerous mature rule-based AFR methods have been applied to CAPP systems. However, these methods are not always fast, accurate, or robust due to the following drawbacks:

(i)  Many rule-based AFR methods use computationally intensive algorithms, such as graph-based methods and volume decomposition methods, resulting in inefficient machining feature recognition.
(ii)  The frequent intersection and penetration of machining features often disrupt the topological structure of original features. This limitation results in poor performance of rule-based AFR methods in recognizing intersecting features, which is also considered one of the most significant challenges in AFR technology.
(iii)  Predefining reliable rules for various machining feature structures is laborious and tedious. Additionally, a single rule system has limited capability in recognizing diverse machining feature categories, making rule-based AFR methods lack flexibility and versatility.

Many studies have combined several types of rule-based AFR methods to absorb the advantages of each method. Examples include the hybrid method of graphs and hints^17^ or combined volumetric decomposition methods and graph-based methods^18^. While these methods have indeed improved the performance of the original methods, they have not fundamentally addressed the problems existing in rule-based methods.

### Learning-based AFR methods

Learning-based AFR methods originated from the rise of ANNs in the 1980s and 1990s^19^ proposed one of the earliest ANN-based AFR methods. This approach encoded the B-Rep solid model into an adjacency matrix of faces including face descriptions and face-face relations, and input it into a five-layer perceptron for machining feature recognition. Hwang’s method^20^ extracted an 8D score vector from the B-Rep model as input to a single-layer perceptron. Based on^20,21^ not only increased the dimension of the score vector to 9D but also used an Adaptive Resonance Theory (ART2) neural network to learn machining feature recognition. Similarly^22^, also used a 9D score vector as input, but his learning framework was a Backpropagation Network (BPN) different from^21^. Additionally^23^, also proposed an ANN-based machining feature recognition method, where the input to the neural network was a 12D score vector that could represent the topological structure and geometric information of machining features. The ANN-based methods laid the foundation for later deep learning methods (mainly deep neural networks). However, due to the immaturity of neural network technology at the time, these methods did not outperform rule-based methods.

FeatureNet^24^ was one of the earliest works in AFR that paid attention to deep learning methods. Reference^24^ proposed a 3D CNN called FeatureNet to learn how to identify machining features from voxelized 3D models. However, FeatureNet could only recognize components with a single feature. For multi-feature components, especially those with intersecting features, it had to rely on watershed segmentation algorithms to divide multiple machining features. This algorithm could only deal with intersecting features with small overlapping areas and had difficulty recognizing highly intersecting features^25^ also proposed a similar segmentation-then-classification approach using 3D CNNs on voxelized models. The difference was that in the segmentation stage, they first used AAG to identify convex and concave machining features of the part, and then used the bounding box method to segment the machining features before voxelization. All these voxel-based methods faced a common trade-off between classification accuracy and computational cost: to improve classification accuracy, the voxel resolution had to be increased, but higher resolution meant computational cost would increase exponentially.

Point clouds have also been applied to AFR, representing a real-world three-dimensional digital description. Reference^26^ learned from the Point Cloud version of CAD models using a modified PointNet. Similarly, for multi-feature components^26^, also adopted^24^’s segmentation-then-classification strategy. However, Ref.^27^ believed that this two-step strategy was complex and time-consuming, because segmentation and classification were performed independently, and the segmented machining features had to go through the neural network multiple times. To address this, they proposed a novel multi-task network, called associatively segmenting and identifying network(ASIN), for machining feature recognition. This network could complete three tasks: clustering similar faces from the part into machining features with unidentified types; predicting the semantic category of each face to determine the type of each machining feature; and identifying the bottom face of the machining features. Experiments showed that ASIN could recognize intersecting features well. It is worth noting that point cloud methods also face the trade-off between classification accuracy and computational cost, which depends on the point cloud density.

Many works approached AFR from a 2D perspective, such as multiple sectional view network (MsvNet) and single shot multibox detector network (SsdNet) proposed by Refs.^28,29^, which used 2D CNNs to learn 2D views of the 3D part models from different angles. However, 2D-view based methods lost the geometric and topological information of 3D part models and even had difficulty accurately locating the machined surfaces. These approaches, MsvNet and SsdNet, were also essentially two-step strategies.

Recently, researchers have refocused attention on graph-based AFR methods but abandoned subgraph matching algorithms in favor of GNNs for processing graph data. References^30,31^proposed a hierarchical B-Rep shape representation that could encode both surface geometry and face topology of the B-Rep, and designed a GNN called Hierarchical CADNet to learn this new shape representation. This network could achieve very high recognition rates even for intersecting features. Reference^30^ represented a true one-step recognition method because Hierarchical CADNet did not require various segmentation operations on the 3D model. Instead, it predicts the feature type for each B-Rep face, a feat not achieved by previous deep learning methods. However, it also had many problems, such as i) The hierarchical graph representation not only occupied a large memory but also slowed down the training of the neural network; ii) Hierarchical CADNet did not consider edge attributes; iii) Whenever a new feature appeared, the neural network had to be retrained from scratch, a common shortcoming not mentioned in previous deep learning methods.

### Graph neural network

Graph Neural Networks (GNNs) have emerged as a powerful tool for pattern recognition and information mining within graph data structures. Since their inception in 2004^32^, GNNs have found applications across a variety of domains, including social networks, recommender systems, traffic forecasting, and biomedicine. The evolution of GNNs has led to the development of advanced variants such as graph convolutional networks^33^, graph attention networks^34^, and GraphSAGE^35^, each enhancing neighborhood aggregation and thereby, the quality of embedding vectors.

The foundational principle guiding the advancement of GNNs draws inspiration from the Weisfeiler-Lehman graph isomorphism test (WL test)^36^, a method pivotal for assessing topological identity between graphs. This analogy with the WL test, which iteratively updates node attribute vectors through neighbor aggregation, has propelled a deeper understanding of how aggregation methods influence GNN performance. In light of these insights, Ref.^3^ introduced the Graph Isomorphism Network (GIN), a model recognized for its exceptional expressiveness in learning graph data.

Despite these advancements, GNNs encounter significant challenges in scaling to greater depths, primarily due to vanishing gradients and excessive smoothing issues. These challenges limit the embedding representations’ diversity as the network deepens, with most cutting-edge GNN models peaking at around four layers before experiencing a marked decline in performance. Addressing these limitations, Ref.^37^ explored solutions from the convolutional neural network (CNN) domain, specifically Residual Connections (Res)^38^, Dense Connections (Dense)^39^, and Dilated Convolution^40^. Through comparative experimentation, it was determined that residual connections offer a promising avenue to surmount depth-related challenges in GNNs.

Building on these developments, this study unveils an efficient and memory-saving Boundary Representation (B-Rep) graph model and introduces Sheet-metalNet, a new Graph Neural Network tailored for machining feature identification. An innovative incremental learning strategy empowers Sheet-metalNet to flexibly recognize new machining features. Additionally, a specialized dataset for sheet metal parts has been developed, broadening the scope of deep learning in Automatic Feature Recognition (AFR). This approach demonstrates the ongoing evolution and potential of Graph Neural Networks in enhancing computational intelligence in manufacturing and beyond.

## Methodology

### B-Rep in graph form

The topological relationships of B-Rep models can be described using graph structures, and graph-based methods are considered one of the most successful methods in rule-based AFR approaches. It converts the B-Rep model into an AAG composed of nodes and arcs. In AAG, nodes correspond to the faces of the model, arcs correspond to the edges of the model and represent connections between faces. Attributes are attached to arcs: if the attribute value is 0, there is a concave connection between two adjacent faces; if the attribute value is 1, there is a convex connection. The method predefines subgraphs of AAG for features and then searches the overall AAG to match the subgraphs. If the corresponding subgraph is found, it is identified as that feature. However, there are two problems with this method: first, subgraph matching is a non-deterministic polynomial-hard (NP-hard) problem, resulting in the high computational complexity of the method; second, the method faces difficulties in dealing with intersecting features. Although graph-based methods have limitations, it does not imply that the graph structure of B-Rep models lacks research value.

**Figure 1:**
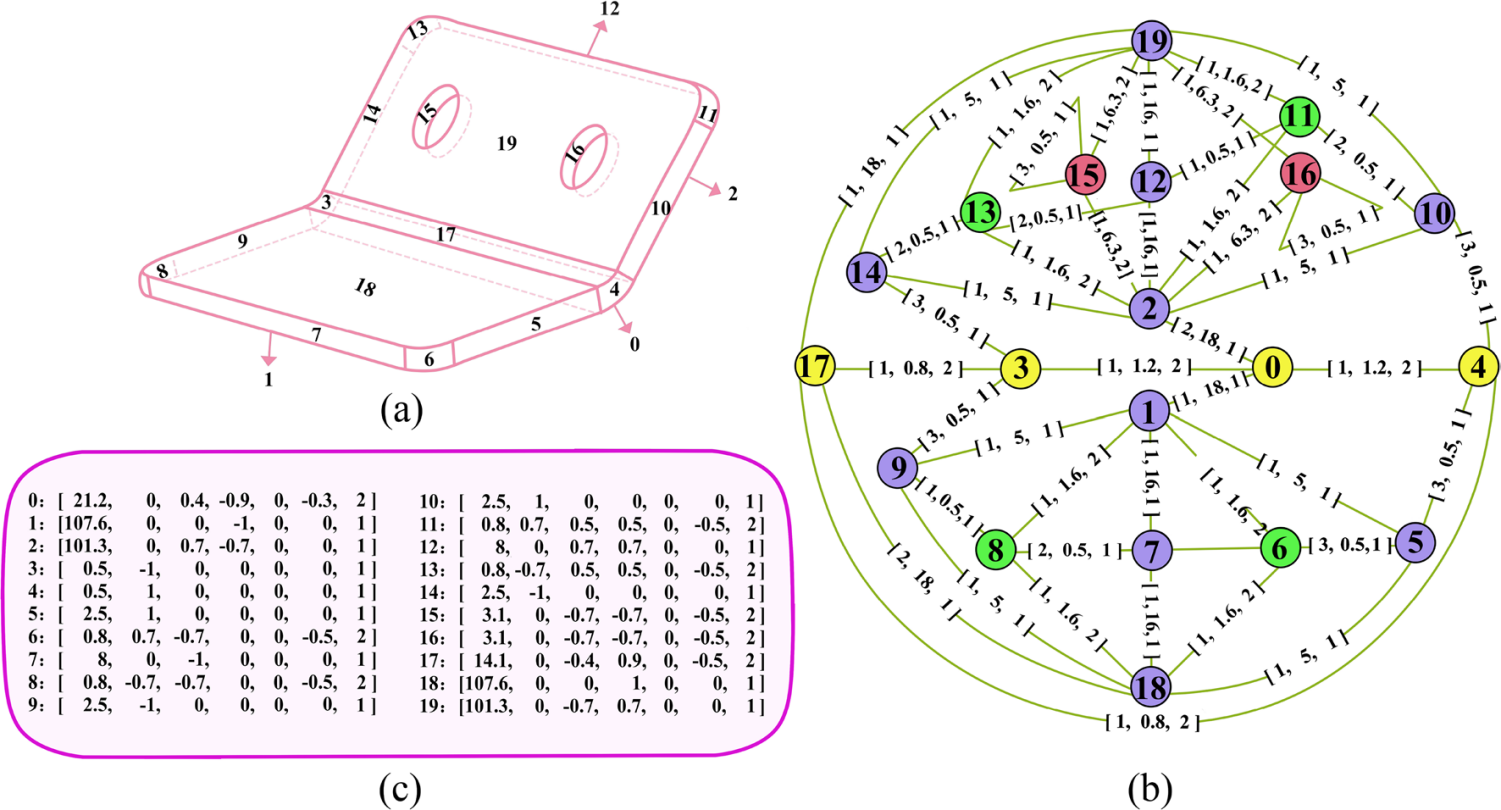
Schematic diagram of a part and its maFEG: (**a**) 3D shape of the part; (**b**) Schematic representation of maFEG diagram for the part; and (**c**) Attribute vectors of each node in (**b**).

Inspired by traditional graph-based automatic feature recognition methods and incorporating recent advancements in graph neural network technologies, a Sheet-metalNet is proposed, which can accurately predict the machining feature class to each B-Rep face of a CAD model belongs to. Furthermore, a Multidimensional Attributed Face-Edge Graph (maFEG), $G=(\nu ,\epsilon ,\chi ,\gamma )$, is established as the mapping of CAD model within the graph domain, drawing on AAG (Fig. 1), and input into Sheet-metalNet for learning, where $\nu$ and $\epsilon$ represent the sets of nodes and edges respectively and $\chi$ and $\gamma$ represent vector spaces constituted by node attributes and edge attributes, respectively. Moreover, $\nu$ and $\epsilon$ are integrated into the adjacency matrix $A\in \mathbb {R}^{n\times n}$, where *n* is both the number of nodes and the number of B-Rep faces within the CAD model. Any element in A can be expressed as follows:

1 $$\begin{aligned} A_{ij}={\left\{ \begin{array}{ll} 1 &{}\text {if edge between i and j},\\ 0 &{}\text {otherwise}. \end{array}\right. } \end{aligned}$$

Based on Eq. (1), $G=(A,\chi ,\gamma )$.

For a single node $\nu _i$, its attribute vector $\chi _i$ has 7 dimensions, which are:

1. The surface area of B-Rep face.
2. X coordinate of the surface normal vector.
3. Y coordinate of the surface normal vector.
4. Z coordinate of the surface normal vector.
5. The average Gaussian curvature of several sampling points on the surface.
6. The average mean curvature of several sampling points on the surface.
7. The surface type (e.g., plane, cylindrical surface, and conical surface), as shown in Table 1.

For a single edge $\epsilon _{ij}$, its attribute vector $\gamma _{ij}$ has 3 dimensions, which are:

1. The convexity (In instances where the angle between the two neighboring faces of an edge exceeds 180^∘^, it is categorized as a convex edge, denoted by a dimension value of 1. Conversely, when the angle between the two neighboring faces falls below 180^∘^, it is characterized as a concave edge, and the corresponding dimension is assigned a value of 2. When the angle between the two adjacent faces measures exactly 180^∘^, it is classified as a flat edge, represented by a dimension value of 3.).
2. The curve length of B-Rep edge.
3. The curve type(e.g., line, circle, ellipse), as depicted in Table 1.

To minimize scale discrepancies across different types of data for easier comparison and processing, linear normalization (min-max normalization) was applied to the numerical values of node and edge attribute vectors before they were fed into the Sheet-metalNet. The formula is expressed as: $Normalized\ Value = (Actual\ Value - Minimum\ Value) / (Maximum\ Value - Minimum\ Value)$

**Table 1:** Surface and curve types in attribute vectors with their assignments.

Surface types	Value	Curve types	Value
Planar	1	Line	1
Cylindrical	2	Circle	2
Toroidal	3	Offset	3
Spherical	4	Hyperbola	4
Conical	5	Ellipse	5
Bezier	6	Parabola	6
B-Spline	7	Bezier	7
Surface of revolution	8	B-Spline	8
Offset	9	Other	9
Surface of extrusion	10		
Other	11		

In contrast to traditional AAG, which focuses only on the topological structure of B-Rep models, maFEG pays attention to both the topological structure and geometric attributes of B-Rep models. This incorporation of both aspects enriches maFEG with a greater amount of information compared to AAG. Additionally, compared to the hierarchical B-Rep graph mentioned in Ref.^30^, maFEG abandons the mesh facet graph level and adds geometric attributes of edges in the B-Rep face adjacency graph level. Overall, the amount of data in maFEG is much less than that of the hierarchical B-Rep graph.

### Representational power of sheet-metalNet

GNNs utilize the adjacency matrix *A* and attribute vectors $\chi$ of nodes in a graph to extract final representation vectors (embeddings) of nodes and graphs. Modern GNNs widely follow a recursive neighbor aggregation strategy, also known as the message passing mechanism, where the process is: each node in a graph iteratively updates its attribute vector by aggregating the attribute vectors of its neighbor nodes. After *k* iterations of aggregation, the node’s attribute vector will capture information about its arbitrary *m*-order ($m\le k$) neighbor nodes within its *k*-hop network range. The above iterative process can be summarized formally as follows:

2 $$\begin{aligned} h_{\nu }^{\left( k\right) }=COMBINE^{\left( k\right) }\bigg (h_{\nu }^{\left( k-1\right) },AGGREGATE^{\left( k\right) }\left( \left\{ h_{\mu }^{\left( k-1\right) }:\mu \in N\left( \nu \right) \right\} \right) \bigg ) \end{aligned}$$

where $h_{\nu }^{\left( k\right) }$ represents the attribute vector of node $\nu$ after the *k*-th iteration, the initialization $h_{\nu }^{\left( k\right) }$, i.e. $h_{\nu }^{\left( 0\right) }=\chi _{\nu }$; $COMBINE^{\left( k\right) }$ and $AGGREGATE^{\left( k\right) }$ are both aggregation functions; $N\left( \nu \right)$ represents the set of all neighbor nodes of node $\nu$.

Xu et al.^3^ posits that the Weisfeiler-Lehman (WL) test sets the upper limit for the representational capabilities of Graph Neural Networks (GNNs) through its injective aggregation update. This injective aggregation allows the WL test to effectively distinguish between different graph structures by mapping distinct node neighborhoods to unique embeddings in the representation space. In the realm of GNNs, if a model incorporates injective aggregation functions similar to those used in the WL test, it has the potential to match the WL test’s discriminative power. In line with this, Sheet-metalNet adopts the Graph Isomorphism Network (GIN), which aligns closely with the principles of the WL test. Its iterative process can be represented by the following formula:

3 $$\begin{aligned} h_{\nu }^{\left( k\right) }=MLP^{\left( k\right) }\bigg (\left( 1+\alpha ^{\left( k\right) }\right) h_{\nu }^{\left( k-1\right) }+\sum _{\mu \in N\left( \nu \right) }h_{\mu }^{\left( k-1\right) }\bigg ) \end{aligned}$$

where $MLP^{\left( k\right) }$ is a multilayer perceptron, which can approximate the injective aggregation function that is desired with arbitrary precision during the network training process, according to the universal approximation theorem; the summation operation in the formula has been proven to be injective; $\alpha ^{\left( k\right) }$ is a learnable parameter used to adjust the weight of node $\nu$’s attribute vector in the summation.

However, Eq. (3) only aggregates node attribute vectors. Considering that maFEG includes not only node attributes but also edge attributes, the complete node attribute iteration update process for Sheet-metalNet involves aggregating the attribute vectors of edges between a given node and its neighboring nodes into the attribute vector of the given node. This results in the following iterative update process for Sheet-metalNet:

4 $$\begin{aligned} h_{\nu }^{\left( k\right) }=MLP^{\left( k\right) }\bigg (\left( 1+\alpha ^{\left( k\right) }\right) h_{\nu }^{\left( k-1\right) }+\sum _{\mu \in N\left( \nu \right) }ReLU\left( h_{\mu }^{\left( k-1\right) }+\epsilon _{\mu ,\nu }\right) \bigg ) \end{aligned}$$

where, *ReLU* is an activation function used for nonlinearization, $ReLU\left( x\right) =\sigma \left( x\right) =max\left( 0,x\right)$; $\epsilon _{\mu ,\nu }$ represents the attribute vector of the edge connecting $\mu$ and $\nu$.

### Development of deep sheet-metalNet

Convolutional Neural Networks (CNNs) have enjoyed remarkable success across various domains, primarily due to the advantages offered by training deep neural networks, which tend to produce more reliable outcomes. Despite their successes, Graph Neural Networks (GNNs) face challenges in achieving similar levels of depth because of vanishing gradients and over-smoothing issues. In response to these challenges, Sheet-metalNet incorporates residual connections to facilitate deeper network architectures.

The so-called residual connection learns an underlying mapping *H* by fitting a residual mapping *F*. The input $G_l$ from the previous layer (layer *l*) of the network is transformed by *F* to become the residual $G_{l+1}^{res}$, which is then added to the unchanged $G_l$ to finally obtain the output $G_{l+1}$ of this layer (layer $l+1$).

5 $$\begin{aligned} G_{l+1}=H\left( G_l,W_l\right) =F\left( G_l,W_l\right) +G_l=G_{l+1}^{res}+G_l \end{aligned}$$

where $W_l$ is the learnable weight parameters of layer *l*.

Combining the node attribute update method in Section "Representational power of Sheet-metalNet" and regularization techniques like Dropout and Normalization, using a pre-activation version, the operation sequence of a residual block is expressed as follows:

6 $$\begin{aligned} Normalization\longrightarrow ReLU\longrightarrow Dropout\longrightarrow Update\_nodes\longrightarrow Res \end{aligned}$$

Connecting several residual blocks forms the backbone network of Sheet-metalNet. After the backbone network finishes iterating, Sheet-metalNet maps the embedding representations of all nodes into logit vectors $\xi$ through a fully connected layer (i.e., performing a linear transformation), where the dimension of $\xi$ is equal to the number of machining feature categories *C* and each component of $\xi$ represents the score of a machining feature category. Finally, the softmax function is used to process the logit vector $\xi$ to obtain the output vector $\hat{y}$, where each component of $\hat{y}$ represents the probability that a node (i.e., a B-Rep face) belongs to a certain machining feature category. For machining feature category *c*, the formula for computing its probability using the softmax function is shown as follows:

7 $$\begin{aligned} \hat{y}_c=\frac{e^{\xi _c}}{\sum _{\kappa =1}^C e^{\xi _\kappa }} \end{aligned}$$

**Figure 2:**
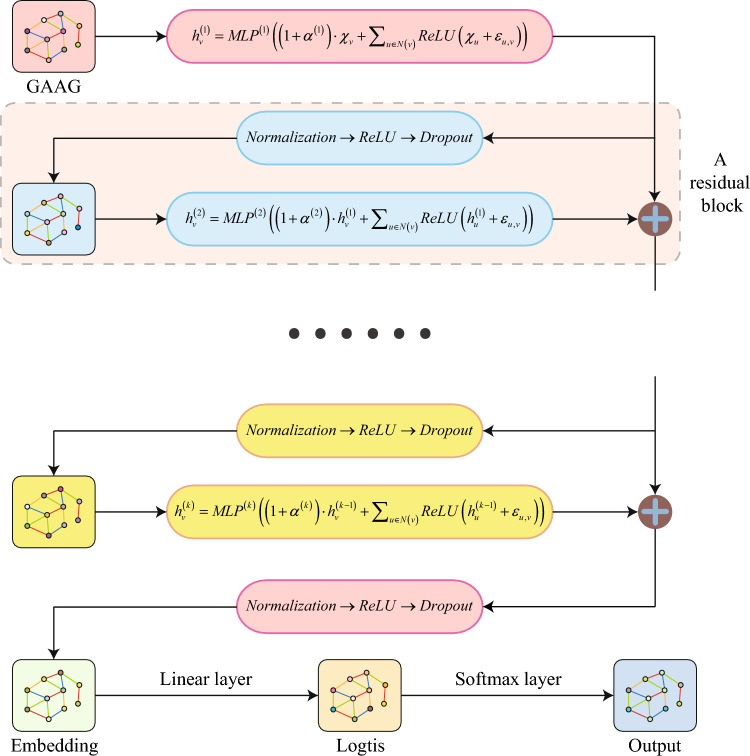
Overall architecture of Sheet-metalNet.

Figure 2 shows the overall architecture of Sheet-metalNet.

### Incremental learning

#### Pretraining and fine-tuning

In practical applications, new components with newly added machining features and different feature intersection patterns continually emerge to adapt to the evolving capabilities of machines and manufacturing processes. Although modern GNNs have very strong generalization capabilities, this generalization ability is premised on the training data and test data being independently and identically distributed. Otherwise, GNNs struggle to provide reasonable responses. It can be affirmed that the emergence of new machining features and new feature intersection forms makes the new data follow a different distribution from the old data. Consequently, these new data instances fall outside the realm of what GNNs can generalize to and, as a result, cannot be recognized by GNNs. Recent research, such as the work by Ref.^41^, also addresses the issue of dynamic adaptability in machine learning, but they focus more on selecting the optimal model rather than optimizing the adaptability of a fixed model.

A straightforward approach would be to mix the new and old data and retrain the neural network from scratch. However, this method will lead to two potential problems: (i) on the one hand, mixing new and old data and retraining requires huge time and space costs; (ii) on the other hand, compared with CAD models of old parts, CAD models of new parts are much smaller in number, leading to class-imbalance problem. In other words, GNNs tend to identify old machining feature categories, causing several new classes to be predicted as old classes. Because of the above problems, incremental learning was introduced. This is a learning paradigm that can continuously and incrementally acquire available information from continuous non-stationary data streams, retaining, integrating, and optimizing old knowledge while absorbing new knowledge. When dealing with the problem of adding new categories in a classification task, it is referred to as class incremental learning.

Class incremental learning usually adopts the pretraining and fine-tuning mechanism in transfer learning^42^ to obtain the optimal initialization parameters. Specifically, the Sheet-metalNet is trained using the pretraining dataset, the trained backbone network is saved as an encoder $g_{\theta }$, and the fully connected layer is saved as a classifier $s_{\theta }$. Class incremental training first builds a similar GNN framework as pretraining (i.e., only the number of categories in the fully connected layer classification head is different), and then directly calls the parameters of $g_{\theta }$ and $s_{\theta }$. To significantly prevent parameters from drifting, a lower learning rate is used during class incremental training to fine-tune $g_{\theta }$ and $s_{\theta }$, as well as to obtain $g_{\theta }'$ and $s_{\theta }'$ which can adapt to the incremental training dataset.

#### Prototype sampling-based replay

The primary challenges of class incremental learning are concentrated in the so-called stability-plasticity dilemma^43^. This dilemma represents a balance between the ability to overcome catastrophic forgetting (i.e., retaining previously learned knowledge) and acquiring new knowledge. Inspired by the storage and recall mechanisms in human memory, Ref.^44^ proposed a replay-based incremental learning method called iCaRL. This method involves sampling a portion of data from previous tasks as exemplars and maintaining a fixed storage area to store these exemplars. Subsequently, the training data of the new classes together with the exemplars are used to update the parameters of the neural network model through the “iCaRL incremental training” algorithm.

The proposed incremental learning strategy references the replay idea of iCaRL and is oriented to graph data. First, given a maFEG, $G=(A,\chi ,\gamma )$, the encoder $g_{\theta }$ can be used to obtain the embedded representations of all nodes as follows:

8 $$\begin{aligned} Z=g_{\theta }\left( A,\chi ,\gamma \right) \end{aligned}$$

For all nodes belonging to a certain category in this graph, the average value of their embedded representations can be achieved as follows:

9 $$\begin{aligned} \varphi _c=\frac{1}{\vert S_c\vert }\sum _{\delta \in S_c}z_\delta \end{aligned}$$

where $S_c$ is the set of all nodes belonging to category *c*, $\vert S_c\vert$ indicates the number of nodes in the set, $z_\delta \in Z$. $\varphi _c$ is used as the prototypical embedding representation of category *c* nodes, named as prototype representation^45^. In statistics, samples often have a higher probability of appearing near their mean. From this perspective, it can be inferred that the embedded representations of the nodes of one category tend to gather around the prototype representation of that category under a given metric space.

**Figure 3:**
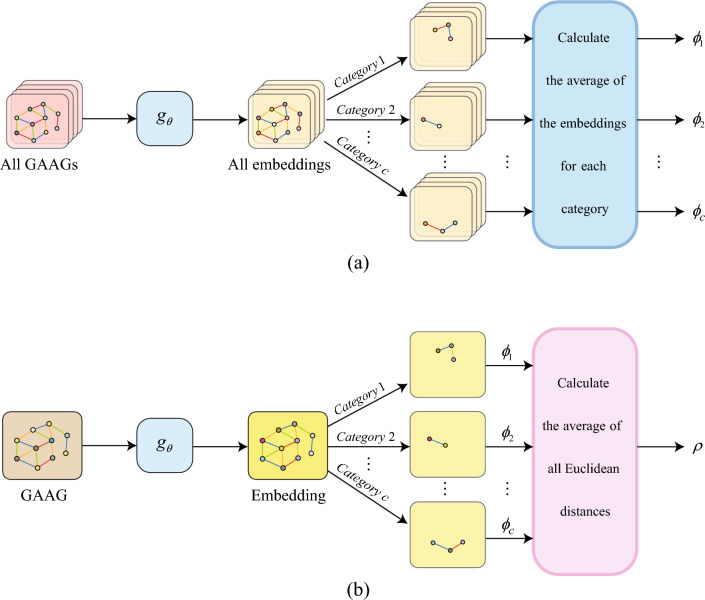
(**a**) Calculation process of prototype representation ${\varphi }_c$; and (**b**) Calculation process of mean distance $\rho$.

Traverse the pretraining dataset and obtain prototype representations of all pre-trained categories using Eq. (9). Then, using the Euclidean distance equation to calculate the distance between the embedded representation of any node $\nu$ in graph *G* and its prototype representation, the average value of this distance for all nodes can be determined as follows:

10 $$\begin{aligned} \rho =\frac{1}{n}\sum _{\nu \in G}\sqrt{\left( z_\nu -\varphi _\nu \right) \cdot \left( z_\nu -\varphi _\nu \right) } \end{aligned}$$

where *n* is the number of nodes in graph *G*, $z_\nu$ and $\varphi _\nu$ are the embedded representation and prototype representation of the node $\nu$, respectively; and $\cdot$ represents the dot product of two vectors. The mean distance $\rho$ characterizes the representativeness of graph *G* in the pretraining dataset. The smaller the $\rho$ value, the more suitable the graph is as an exemplar for class incremental training. Figure 3 depicts the calculation process of prototype representation ${\varphi }_c$ and mean distance $\rho$.

To mitigate the influence of the class-imbalance problem, the principle should also be followed that the number of nodes in the exemplar is roughly equal to the number of nodes in the new dataset. Finally, the graphs from the new dataset and the examples are pairwise concatenated into new graphs and fed into the class-incremental training of Sheet-metalNet for learning.

#### Parameter regularisation based on knowledge distillation

Another potentially effective method to overcome the stability-plasticity dilemma is regularization based on knowledge distillation. Initially^46^, proposed knowledge distillation to transfer information between neural network models of different scales. Subsequently^47^, applied it under the name of Learning without Forgetting (LwF) for class incremental learning to maintain the generalization ability of the old categories in the new and old models of the same neural network.

Knowledge distillation is a type of regularization acting on the loss function: Sheet-metalNet uses the following multi-class cross-entropy loss function to encourage the output vector $\hat{y}$ to be consistent with the ground truth *y*:

11 $$\begin{aligned} L_{hard}\left( y,\hat{y}\right) =-y\cdot \log \hat{y} \end{aligned}$$

where *y* is the one-hot encoded vector of the ground truth labels; while knowledge distillation uses the following KL divergence loss function to constrain the output of the new network model from deviating too far from the output of the old network model, shown as follows:

12 $$\begin{aligned} L_{soft}\left( p,q\right) =-p\cdot \log \frac{p}{q} \end{aligned}$$

where *p* is the corrected output vector of the old network model; *q* is the corrected output vector of the new network model; and the components corresponding to the machining feature category *c* of the two vectors are expressed as follows:

13 $$\begin{aligned} \left\{ \begin{aligned} p_c=\frac{e^{{\omega _c}/{T}}}{\sum _{\kappa =1}^C e^{{\omega _\kappa }/{T}}} \\ q_c=\frac{e^{{\zeta _c}/{T}}}{\sum _{\kappa =1}^C e^{{\zeta _\kappa }/{T}}} \\ \end{aligned} \right. \end{aligned}$$

where $\omega$ and $\zeta$ are the logit vectors of the old and new network models respectively, and *T* is the distillation temperature, which is a hyperparameter.

Adding Eqs. (11) and (12), this study introduces the hyperparameter $\lambda$ to balance their effects, finally obtaining the total loss function for class incremental training as follows:

14 $$\begin{aligned} L=L_{hard}+\lambda L_{soft} \end{aligned}$$

**Figure 4:**
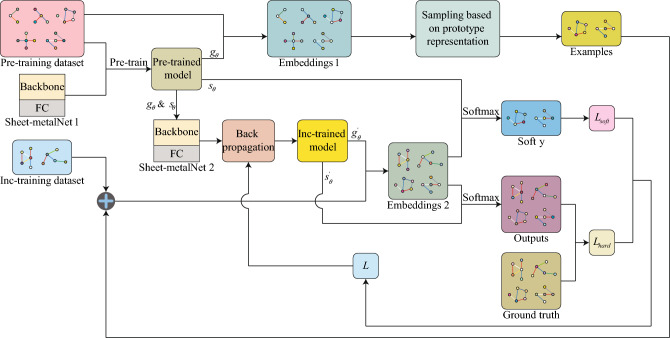
Overall framework of the incremental learning strategy of Sheet-metalNet.

The three methods discussed in Sect. "Incremental learning" from the incremental learning strategy of Sheet-metalNet, which is designed to continuously learn the identification process of the machining features from an expanding dataset of real industrial part CAD models. Figure 4 summarizes the overall framework.

## Dataset creation

### Overview of the SMCAD dataset

Deep learning is data-driven, and almost all successful deep learning methods rely heavily on supervision. Therefore, deep learning-based AFR methods often require large-scale three-dimensional CAD model datasets with labeled machining features to train neural networks. Currently, there are large CAD model datasets available, such as ABC dataset^48^ and MCB dataset^49^, but they do not contain the labels required for machining feature recognition tasks. Customized 3D CAD model datasets are accessible^24,27,30^, but they only considered machined parts. This is because the features on machined parts are mostly machined by milling (i.e. material removal manufacturing), so creating manually synthesizing datasets only involves cutting away the shapes of machining features from stock cubic steel blanks to obtain CAD models. In contrast, sheet metal parts, which involve non-milling processes such as bending, stamping, and riveting, have the characteristics of uniform thickness everywhere and complex and varied spatial structures. This makes the synthesis of CAD models for sheet metal parts challenging, and as a result, there are few 3D CAD model datasets for sheet metal parts.

**Figure 5:**
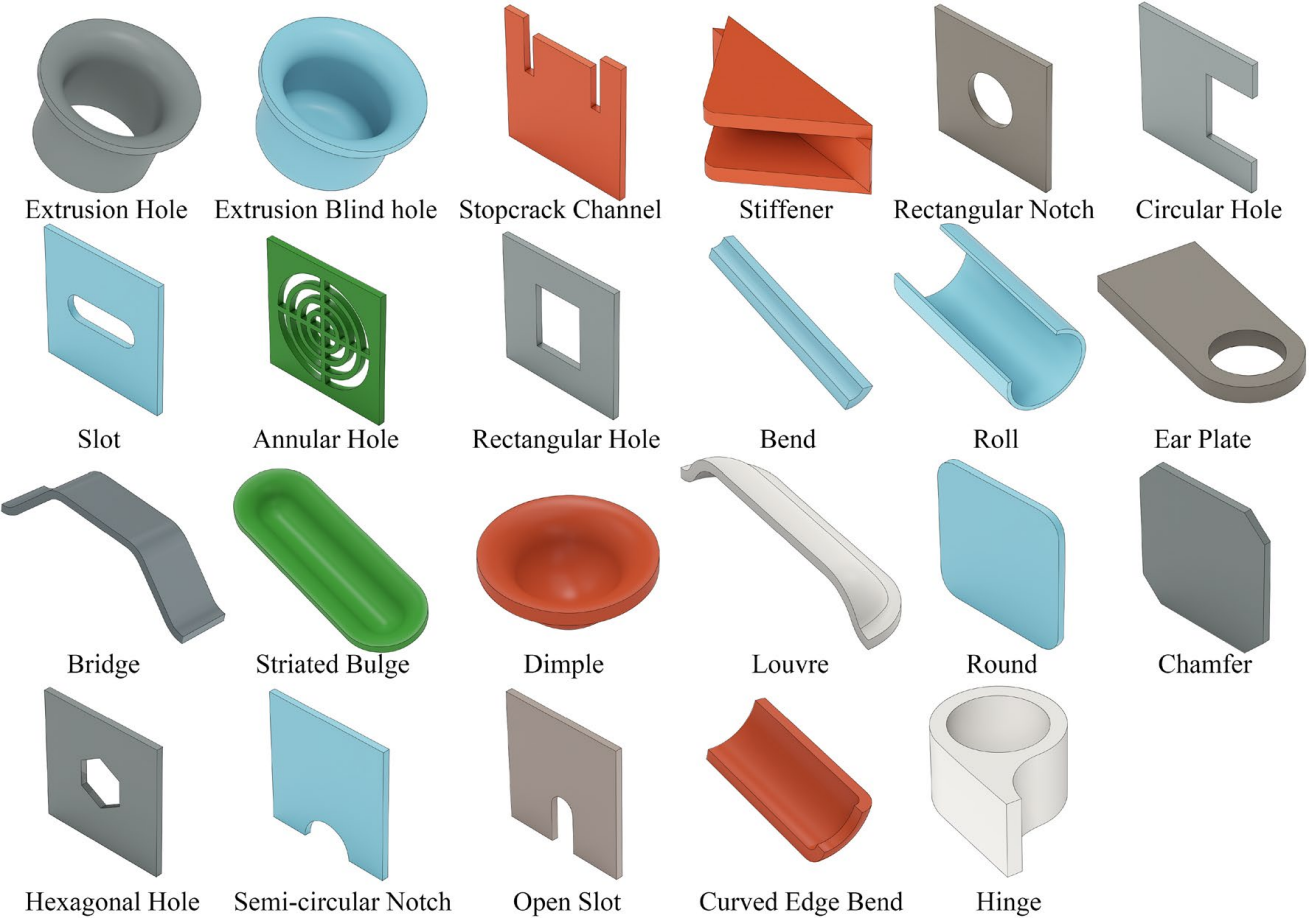
Machining features of sheet metal parts in SMCAD dataset. (The first two rows represent the old machining features in the pretraining dataset, and the last two rows represent the new machining features in the incremental training dataset).

To fill this gap, a 3D CAD model dataset of sheet metal parts represented in B-Rep form, called the SMCAD dataset, was generated using parametric modeling techniques and a random sampling strategy through PythonOCC^50^. The dataset consists of two parts: the pretraining dataset and the incremental training dataset. The pretraining dataset was used to train the neural network to recognize 12 types of sheet metal machining features and primary planes. As usual, the pretraining dataset was divided into three subsets: training set (containing 49,000 CAD models), validation set (containing 10,500 CAD models) and test set (containing 10,500 CAD models). These three subsets were created independently and have no intersection. The incremental training dataset was used to verify the feasibility of the incremental learning strategy. Similarly, it was also divided into three subsets: incremental training set (containing 7,000 CAD models), incremental validation set (containing 3,000 CAD models) and incremental test set (containing 3,000 CAD models). CAD models in the incremental training set only contain 11 new types of machining features, a small number of old machining features and primary planes, while the incremental validation set and test set included all 23 types of machining features and primary planes. Figure 5 shows the old machining features in the pretraining dataset and the new machining features in the incremental training dataset, and Table 2 lists the names of all machining features and their corresponding labels.

**Table 2:** Machining features of sheet metal in SMCAD dataset and their corresponding labels.

Old machining feature	Label	New machining feature	Label
Extrusion hole	0	Bridge	13
Extrusion blind hole	1	Striated bulge	14
Stopcrack channel	2	Dimple	15
Stiffener	3	Louvre	16
Rectangular notch	4	Round	17
Circular hole	5	Chamfer	18
Slot	6	Hexagonal hole	19
Annular hole	7	Semi-circular notch	20
Rectangular hole	8	Open slot	21
Bend	9	Curved edge bend	22
Roll	10	Hinge	23
Ear plate	11		
Principal plane	12		

### SMCAD dataset generation algorithm

**Figure 6:**
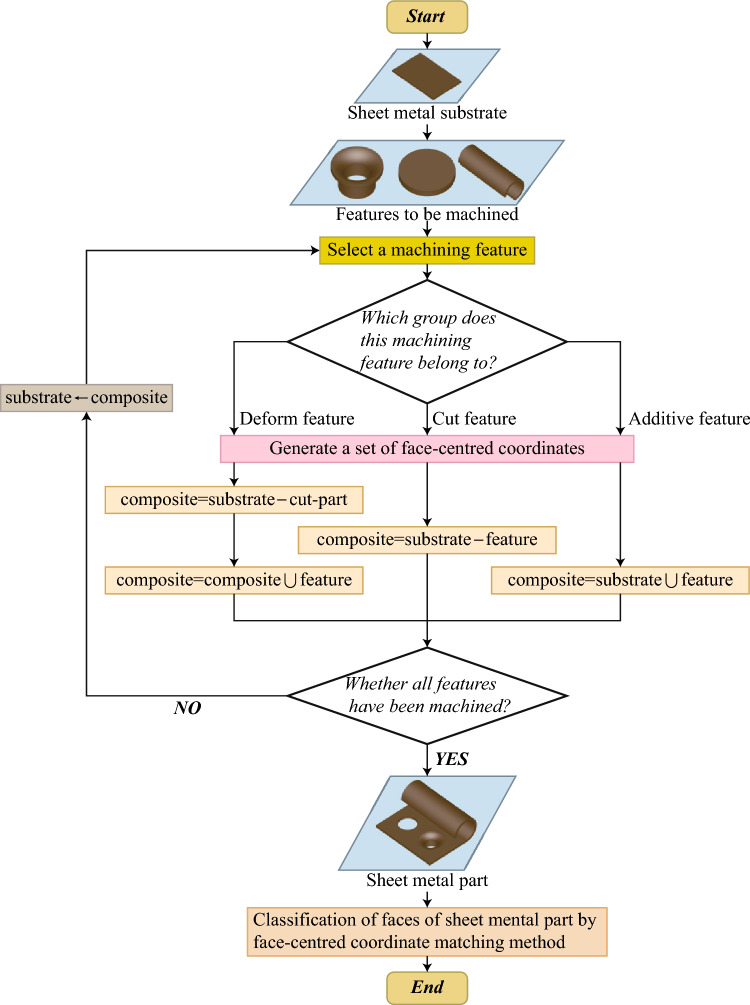
Flowchart of the SMCAD dataset generation algorithm.

**Figure 7:**
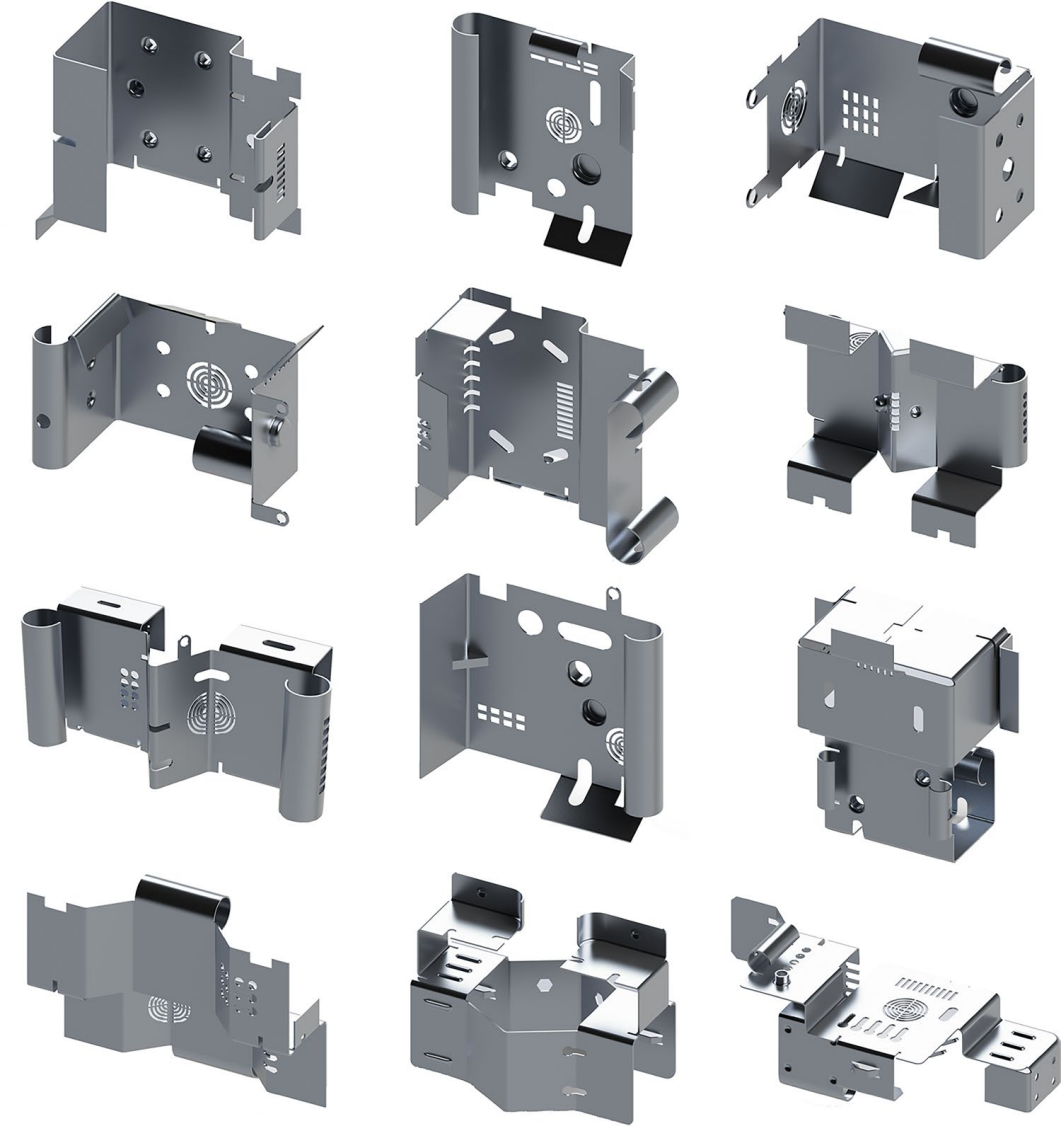
Samples from the SMCAD dataset.

In this study, the SMCAD dataset was created using the following algorithm (Fig. 6):

1. Generate the base models of sheet metal parts, named sheet metal substrates. A sheet metal substrate consists of primary planes, bends, and rolls. SMCAD generates various sheet metal substrates with different shapes. The sheet metal substrates used in the training set cannot be included in the test set or validation set.
2. Create parameterized models for all machining features. Each parameterized model is defined as a Class, and the 23 models are divided into three groups: (i) Deform features (including Extrusion hole, Extrusion blind hole, stopcrack channel, stiffener, bridge, striated bulge, dimple, louvre). These features are relatively complex. When combined with the sheet metal substrate, a Boolean cut operation is initially performed to cut out the shape of the Deform feature on the sheet metal substrate, and then a Boolean union operation is performed to merge the remaining part of the Deform feature into the combined shape. (ii) Cut features (including rectangular notch, circular hole, slot, annular hole, rectangular hole, round, chamfer, hexagonal hole, semi-circular notch, open slot). These features only require Boolean cut operations when combined with the sheet metal substrate. (iii) Additive features (bend, roll, ear plate, curved edge bend, hinge). These features only require Boolean union operations when combined with the sheet metal substrate.
3. Input geometric and positional parameters to all parameterized models to generate a machining feature library. To avoid interference between features or duplicate combined shapes, and to facilitate attaching feature labels to the combined shapes afterward, the geometric parameters and positional parameters are planned when generating individual shapes. Each feature is defined with several shapes of different sizes and positions, and then all shapes are placed within the same spatial context. Subsequently, their sizes and positions are adjusted to refine their boundaries, to avoid any overlap among features.
4. Randomly sample several machining features from the feature library and randomly select one shape of each machining feature to combine with the sheet metal substrate. Additionally, traverse each B-Rep face of the machining feature to generate the set of centroids of B-Rep faces for this feature.
5. Determine the group each machining feature belongs to. If it is a deform feature, the combination order is to perform the Boolean cut operation first and then the Boolean union operation. If it is a cut feature, it directly performs the Boolean cut operation; if it is an additive feature, it directly performs the Boolean union operation.
6. Assign labels corresponding to the machining feature for each B-Rep face of the combined shape. Due to the setting in Step (3), there is no overlap in the spatial positions of different types of features. By traversing each B-Rep face of the combined shape and matching its centroid coordinates with the centroid coordinate collection of each feature from Step (4), the category of each B-Rep face is determined, and the corresponding label is assigned.
7. After all operations are completed, change the sheet metal substrate and repeat Steps 1) to 6). All generated sheet metal CAD models are finally stored in the SMCAD dataset as .step files. Figure 7 shows examples of the sheet metal CAD models in the SMCAD dataset.

## Experimental results and discussion

### Experimental settings and feature recognition results

Sheet-metalNet was developed using PyTorch Geometric library^51^, which is a deep learning framework for non-Euclidean data. The backbone network of Sheet-metalNet consists of 13 residual blocks stacked together. Each residual block employed the aggregation Eq. (4), which uses a two-layer MLP with 256 neurons in both the hidden and output layers. The dropout rate between residual blocks was 0.2. ADAM optimizer was used to optimize the learnable parameters of the encoder (backbone network) and classifier (fully connected layer). The initial learning rate was set to 0.0005 and the weight decay was set to 0.0005. It should be emphasized that Sheet-metalNet was a general mechanical part feature recognition network. Therefore, the performance of Sheet-metalNet was validated using both the SMCAD dataset and the publicly available MFCAD++ dataset^30^ as benchmark datasets. These graph data were batched and fed into Sheet-metalNet, where each batch contained different numbers of maFEGs, and the total number of nodes of all maFEGs did not exceed 10,000. Additionally, all experiment codes were run on an NVIDIA GeForce RTX 2080Ti GPU.

**Figure 8:**
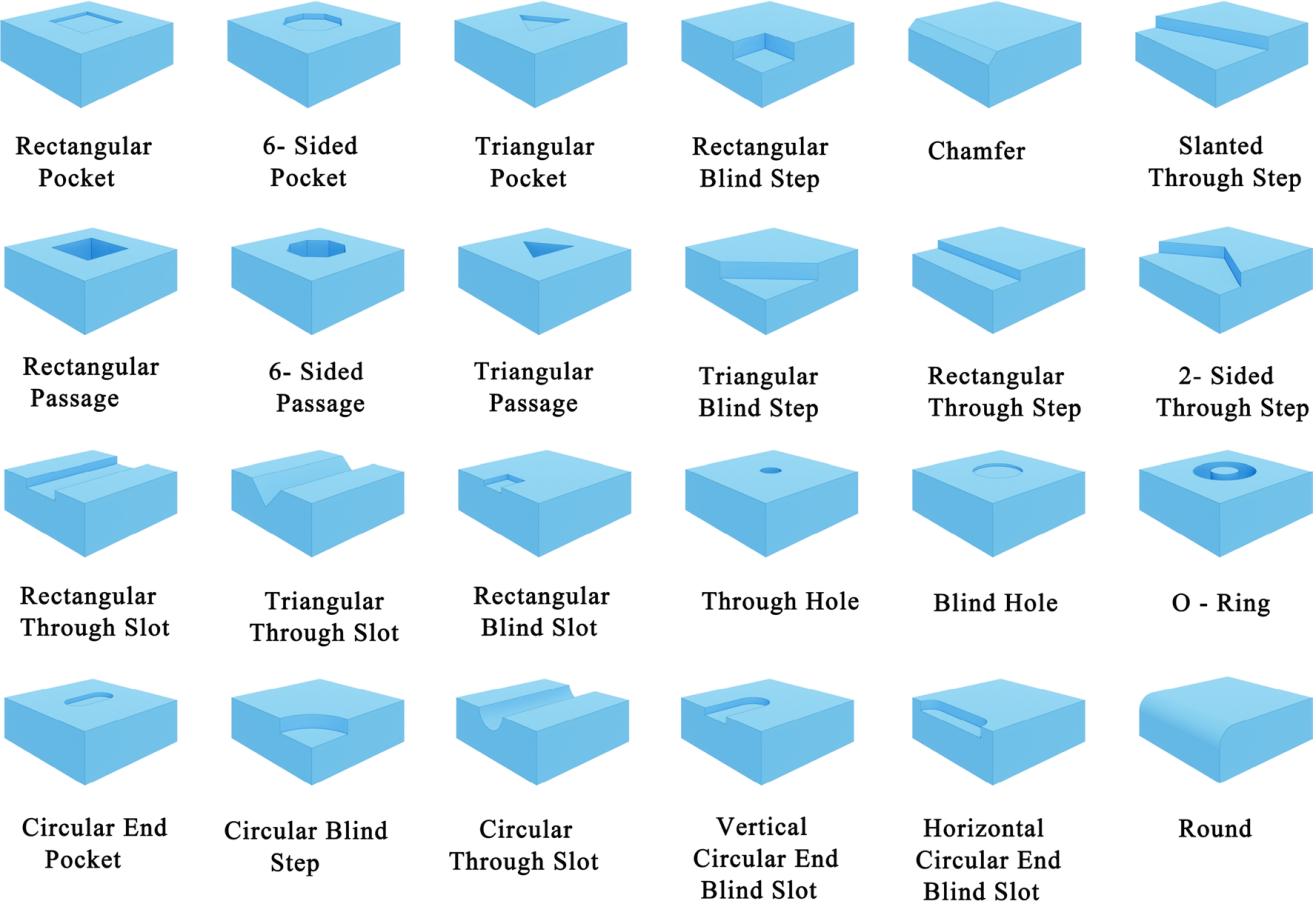
Machining features of the MFCAD++ dataset^30^.

MFCAD++ dataset consisted of 59,655 CAD models, which were split into training, validation, and test sets with a ratio of 70:15:15. Each CAD model contained 3 to 10 machining features, with rich intersecting features arising from their interactions. All machining feature categories are shown in Fig. 8. Colligan et al.^30^ provided two state-of-the-art machining feature recognition networks Hierarchical CADNet (Adj) and Hierarchical CADNet (Edge). Together with Sheet-metalNet, they were each trained on the MFCAD++ dataset for a fixed number of 100 epochs, with each epoch involving a pass of the entire training set through the network. Additionally, the recognition accuracy on the validation set, defined as the ratio of correctly classified B-Rep faces to the total number, was calculated for each epoch as the metric to select the optimal network model.

**Figure 9:**
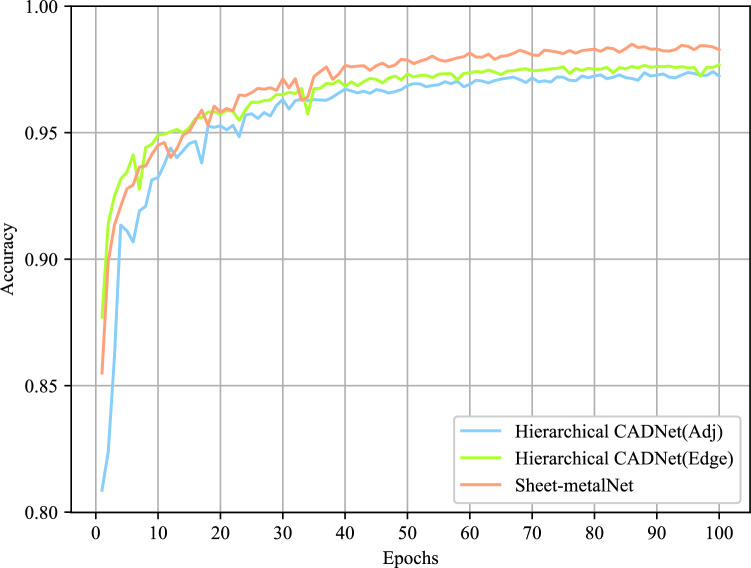
Evolution of recognition accuracy on MFCAD++ validation set over epochs. (The convergence interval is defined as the accuracy range corresponding to the flat area of the curve).

**Figure 10:**
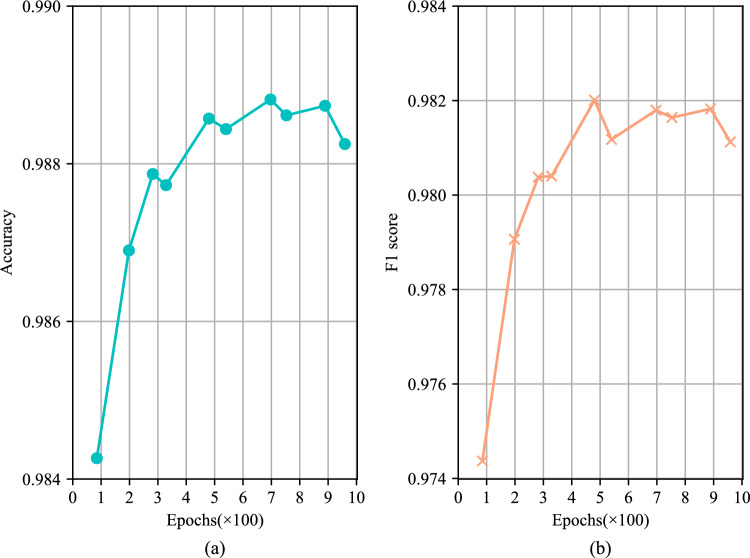
Exploring the performance limit of Sheet-metalNet on MFCAD++ dataset.

The network model with the highest validation accuracy was used to predict machining feature categories of all CAD model B-Rep faces in the test set. The accuracy, macro F1 score, and average time per epoch during training were recorded as evaluation metrics. The results are shown in Table 3. The results indicated that Sheet-metalNet not only achieved higher accuracy and F1 score on the MFCAD++ test set compared to Hierarchical CADNet (Adj) and Hierarchical CADNet (Edge), but it also trained significantly faster than the two versions of Hierarchical CADNet. The notable improvement in training speed could be attributed, in part, to the dataset size difference. The hierarchical B-Rep graph also contains refined mesh information, making its data volume much larger than maFEG. Additionally, the choice of the learning framework might play a role; Hierarchical CADNet used TensorFlow 2, which was not a specialized framework for handling graph data. To provide further insight into the training progress, Fig. 9 shows the validation accuracy curves over epochs of the networks. It displays that after 40 epochs, the validation accuracy curves of all networks tended to be stable, indicating that they reached the convergence interval, and Sheet-metalNet’s convergence interval was higher than the other two networks. Additionally, to explore the performance limit of Sheet-metalNet on the MFCAD++ dataset, the total number of epochs was increased to 1000, and the test accuracy and F1 score of the best model were recorded every 100 epochs (Fig. 10). When the number of epochs reached 480, the test accuracy and F1 score no longer improved significantly, indicating Sheet-metalNet reached its performance limit. At this point, the test accuracy and F1 score were 98.86% and 98.20%, respectively.

**Table 3:** Experimental results on MFCAD++ dataset.

Network	Accuracy (%)	F1 score (%)	Average time to iterate an epoch(s)
Hierarchical CADNet (Adj)	97.27	96.34	432.44
Hierarchical CADNet (Edge)	97.62	96.86	483.22
Sheet-metalNet	98.43	97.44	10.14

**Figure 11:**
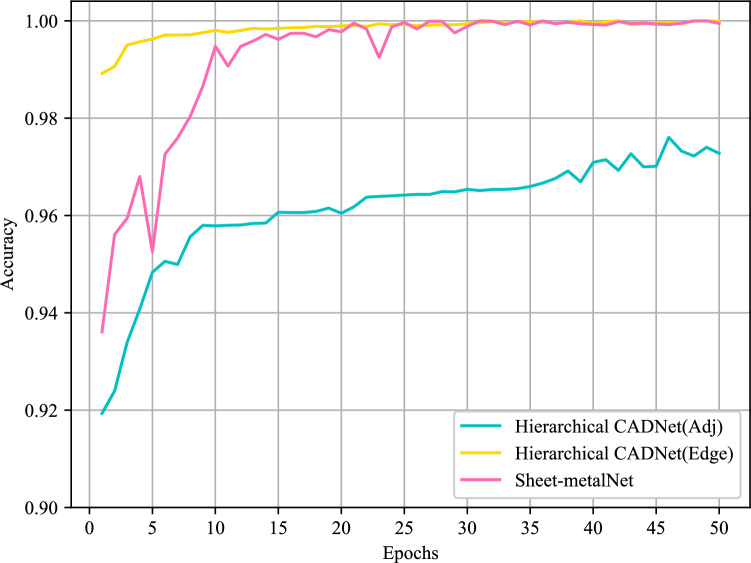
Evolution of recognition accuracy on SMCAD pretraining validation set over epochs. (All networks enter the convergence interval after 20 epochs).

**Figure 12:**
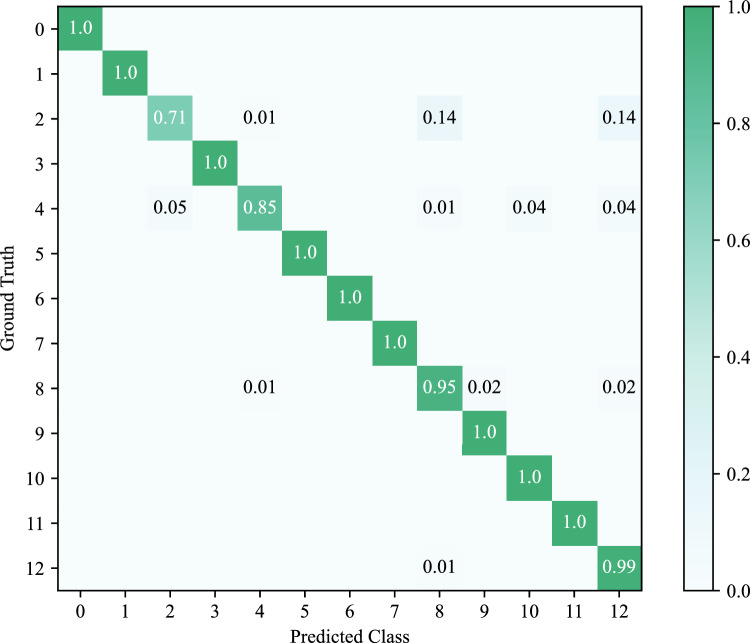
Confusion matrix of Hierarchical CADnet (adj) predictions on SMCAD pretraining test set.

While maintaining the hyperparameters for each network, the study repeated the “train-validate-test” experiments using the SMCAD pretraining dataset. Since the training speed of Hierarchical CADNet was too slow, the number of epochs for all networks was set to 50. Table 4 gives the experimental results. In terms of performance, the accuracy and F1 score of recognizing machining features by Sheet-metalNet and Hierarchical CADNet (Edge) exceeded 99%, much higher than Hierarchical CADNet (Adj). In terms of training efficiency, Sheet-metalNet was undoubtedly superior to the two versions of Hierarchical CADNet. Similar to previous experiments, the validation accuracy curve in Fig. 11 was plotted. However, this curve gave an unfavorable conclusion that the convergence speed of Hierarchical CADNet (Edge) on the validation set was significantly faster than Sheet-metalNet. To explain this phenomenon, the confusion matrix of Hierarchical CADNet (Adj) experimental results is shown in Fig. 12. Hierarchical CADNet (Adj) struggled to recognize 3 maching features: stopcrack channel, rectangular notch, and rectangular hole, as their topological structure and geometric information, were significantly similar. Compared with Hierarchical CADNet (Adj), Hierarchical CADNet (Edge) split the adjacency matrix into convex edge adjacency matrix, concave edge adjacency matrix, and flat edge adjacency matrix, which means that the above three machining features were artificially distinguished at the beginning of training. However, maFEG implicitly incorporated the convexity and concavity of edges into the attribute vector, which caused Sheet-metalNet to require several epochs to learn the information of convexity and concavity of edges.

**Table 4:** Experimental results on SMCAD pretraining dataset.

Network	Accuracy (%)	F1 score (%)	Average time to iterate an epoch(s)
Hierarchical CADNet (Adj)	97.62	96.74	4397.87
Hierarchical CADNet (Edge)	99.99	99.98	4709.31
Sheet-metalNet	99.99	99.99	81.33

### Why Sheet-metalNet works

Sheet-metalNet relies on the most primitive GNN architecture and introduces 3 measures: maFEG, GIN aggregating edge attributes, and residual connections to improve the performance of the original GNN. The purpose of this section was to explore the impact of maFEG attribute vectors $\chi$ and $\gamma$, aggregation functions, and residual connections on the overall performance of Sheet-metalNet. This exploration aimed to provide insights into why Sheet-metalNet was effective. Since the training speed of Sheet-metalNet on the MFCAD++ dataset was much faster than on the SMCAD pretraining dataset (Tables 3 and 4), the study chose to experiment on the MFCAD++ dataset. Additionally, all unrelated variables of Sheet-metalNet (e.g., learning rate and dropout rate) remained consistent with Sect. "Experimental settings and feature recognition results". The number of iterations was fixed at 100 epochs, and the accuracy and F1 score on the test set were still used as evaluation metrics for the overall performance of the network.

**Figure 13:**
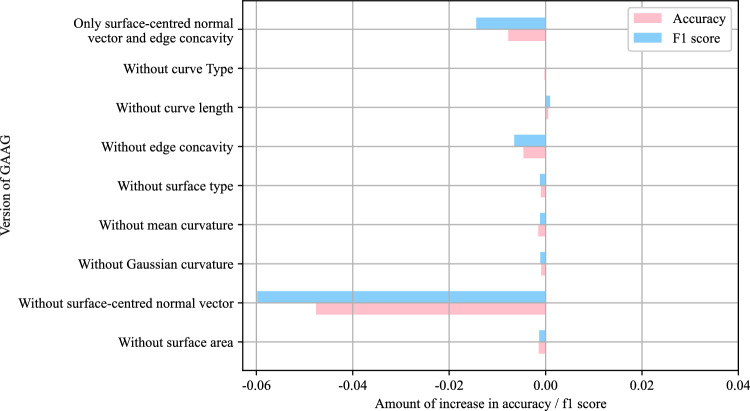
Histogram of attribute vector ablation experiment results.

First, for maFEG attribute vectors $\chi$ and $\gamma$, a series of ablation experiments were conducted, removing one node attribute (the three coordinates of the surface normal vector were considered one attribute) or one edge attribute each time. The experimental results were plotted as the horizontal bar chart shown in Fig. 13, where the horizontal axis is the change in accuracy and F1 score after removing an attribute from maFEG. It can be seen that when the surface normal vector was removed from vector $\chi$, the accuracy and F1 score decreased significantly; when the convexity and concavity was removed from vector $\gamma$, the accuracy and F1 score also decreased to a certain extent. This shows that the above two attributes had the greatest impact on the overall performance of Sheet-metalNet, or in other words, these two attributes were the most important in maFEG. Although the remaining 6 attributes had little individual impact on Sheet-metalNet performance, when all 6 attributes were removed, the accuracy and F1 score also decreased significantly. This suggests that Sheet-metalNet was not very sensitive to a single attribute of maFEG. It might learn the combination patterns and correlation patterns between attributes. Therefore, it could not be concluded that these 6 attributes are unimportant or negligible.

**Figure 14:**
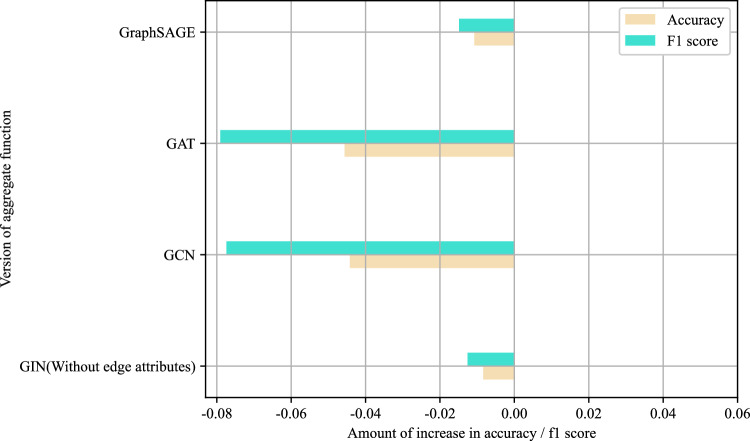
Histogram of aggregation function comparison experiment results.

Secondly, for the aggregation function, another 4 mainstream GNN aggregation functions were selected for comparison with GIN aggregating edge attributes, including GCN, GAT, GraphSAGE, and GIN without aggregating edge attributes. The experimental results (Fig. 14) reveal that Sheet-metalNet using GAT and GCN performs poorly because these two aggregation functions lacked distinction for maFEG encoding. In contrast, GraphSAGE and GIN without edge attribute aggregation exhibited better performance, although they still fell short of the excellent results achieved by GIN with edge attribute aggregation, highlighting the importance of edge attribute information in maFEG. Notably, GraphSAGE in the experiment did not aggregate edge attributes. Theoretically, the original GIN aggregation function in Sheet-metalNet could be replaced with GraphSAGE, provided that GraphSAGE was redesigned to incorporate edge attribute aggregation for maFEG, although specific design details are not discussed here.

**Figure 15:**
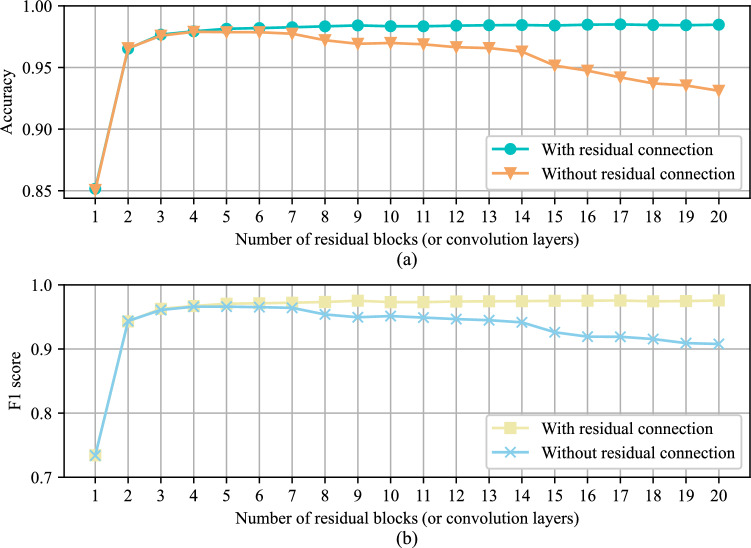
Accuracy and F1 score curves of Sheet-metalNet with different numbers of layers.

Finally, for the residual connection mechanism, Sheet-metalNet without using residual connections was developed. The original Sheet-metalNet and Sheet-metalNet without residual connections with layer numbers from 1 to 20 were trained respectively, and the accuracy and F1 score on the test set of networks with different numbers of layers were recorded and plotted as Fig. 15. As the number of layers increased, the performance of the original Sheet-metalNet gradually improved. The optimal performance was achieved when the network had 17 layers, resulting in accuracy and F1 score of 98.49% and 97.56%, respectively. In contrast, Sheet-metalNet without residual connections experienced a gradual decline in performance after surpassing 4 layers. At 4 layers, the highest accuracy and F1 score of Sheet-metalNet without residual connections were only 97.90% and 96.59%. The experimental results show that: (i) residual connections can indeed solve the problems of gradient vanishing and over-smoothing, improving the depth of Sheet-metalNet; (ii) the deeper Sheet-metalNet performs better, but after about 9 layers, the improvement of Sheet-metalNet performance with depth is no longer significant.

### Necessity and feasibility of incremental learning

**Figure 16:**
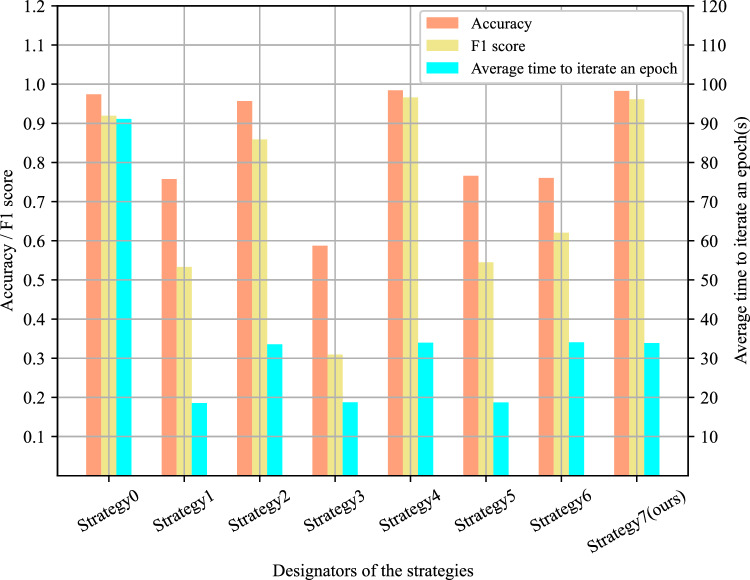
Histogram of comparison results for incremental learning strategies.

In this section, the most naive approach termed the “direct mixing strategy,” where new and old data are combined, and the neural network is retrained from scratch. This strategy serves as the baseline. In contrast, 3 incremental learning methods proposed in Sect. "Incremental learning" constitute the proposed incremental learning strategies. Incremental training sub-datasets are also created in the SMCAD dataset to verify the necessity and feasibility of the proposed incremental learning strategies. The size of the incremental training datasets is much smaller than the size of the pre-training datasets, reflecting the scarcity of samples for new machining features in real industrial scenarios. Additionally, the 3 incremental learning methods are combined in different ways to form another 6 different strategies for comparison with the direct mixing strategy and the incremental learning strategies. The experimental results are shown in Fig. 16. The details of each strategy corresponding to the designators in Fig. 16 are provided in Table 5.

**Table 5:** Designators and details of different strategies.

Designator	Strategy details
Strategy0	Direct Mixing
Strategy1	Pre-training and fine-tuning
Strategy2	Replay-based
Strategy3	Knowledge distillation
Strategy4	Pre-training and fine-tuning + Replay-based
Strategy5	Pre-training and fine-tuning + knowledge distillation
Strategy6	Replay-based + knowledge distillation
Strategy7(ours)	Pre-training and fine-tuning + Replay-based + knowledge distillation

**Figure 17:**
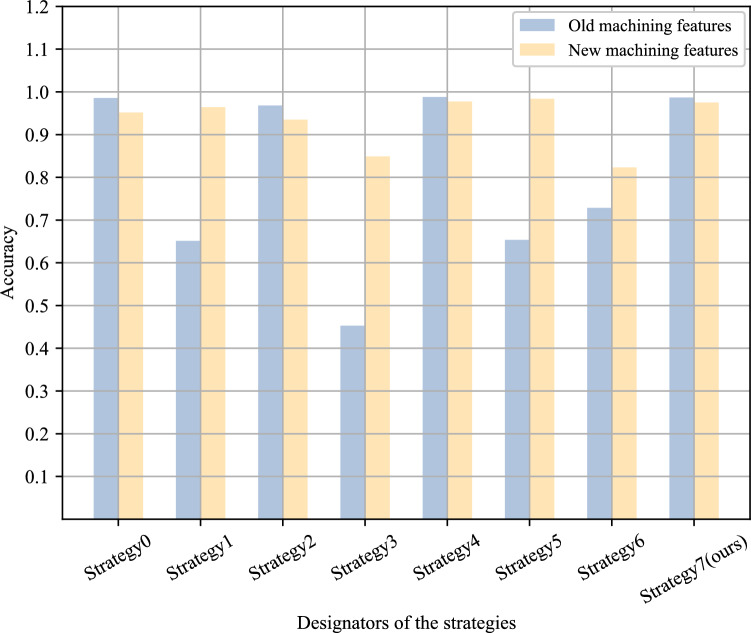
Histogram of new and old machining feature recognition accuracy for different strategies.

In Fig. 16, the accuracy and F1 score of Strategy4 are the highest, reaching 98.43% and 96.60%, respectively. The accuracy and F1 score of Strategy7 are the second highest, reaching 98.28% and 96.18% respectively. The only difference between the two is that Strategy7 has an additional knowledge distillation step compared to Strategy4. At the same time, strategies with knowledge distillation steps like Strategy3, Strategy5, and Strategy6, did not achieve the expected results. Furthermore, the recognition accuracy of new and old machining features were aggregated for all strategies, as shown in Fig. 17. Strategy1, Strategy3, and Strategy5 exhibited significantly lower accuracy in recognizing old machining features compared to new ones, indicating varying degrees of catastrophic forgetting. Among them, Strategy3 with only knowledge distillation experienced the most severe impact. In comparison, Strategy2, Strategy4, and Strategy7,s effectively avoided catastrophic forgetting and demonstrated decent learning capabilities on new machining features. This analysis underscores that, among the 3 components of the Sheet-metalNet incremental learning strategy, the replay component yields the best results. The pretraining-fine-tuning step also contributes positively, while the knowledge distillation component is notably less impactful. Its primary limitation is its inability to effectively address the issue of catastrophic forgetting.

**Figure 18:**
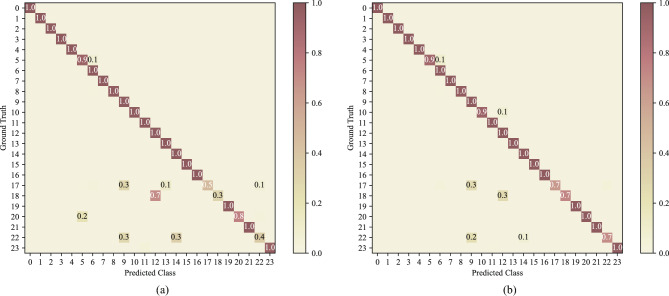
Confusion matrices for: (**a**) Strategy0’s predictions on the incremental test set; (**b**) Strategy4’s predictions on the incremental test set.

For the baseline Strategy0, it exhibited the longest time per epoch, exceeding 90 s. Its accuracy and F1 score are also lower than Strategy4 and Strategy7. As shown in Fig. 17, the accuracy and F1 score differences between them are mainly on recognizing new machining features. Figure 18 shows the confusion matrices on the incremental test set for Strategy0 and Strategy4. Compared to Strategy4, Strategy0 incorrectly classified numerous new machining features as old ones. This is attributed to the class-imbalance problem mentioned in Sect. "Incremental learning". In Strategy0, the substantial difference in the quantity of new and old feature samples led to network inertia in predicting old feature B-Rep faces. Fundamentally, Strategy4 can be regarded as a variant of direct mixing, but its data sampling approach based on prototype representations not only reduces training time and costs but also mitigates class imbalance issues and enhances the accuracy of new machining feature recognition.

## Conclusion

This study proposes a graph structure language maFEG to describe the topological structure and geometric information of mechanical part CAD models. It also introduces a GNN called Sheet-metalNet to learn potential patterns of machining features in maFEG. Sheet-metalNet can effectively encode maFEG and accurately classify nodes (i.e. B-Rep faces). Through a series of experiments, Sheet-metalNet was compared with Hierarchical CADNet in terms of recognition accuracy, F1 score, and training speed on MFCAD++ and SMCAD datasets. The experimental results show that the proposed method has better performance and higher efficiency.

Furthermore, to address the limitation of deep learning algorithms in dynamically recognizing new machining features, an incremental learning strategy was introduced. Assuming the existence of a dynamically expanding real industrial part CAD model dataset, this strategy can help Sheet-metalNet retain recognition capabilities on old machining features while improving recognition of new machining features, even as the dataset slowly grows in size. Relevant experiments indicated that the strategy performance and efficiency surpassed the simplest direct mixing strategy.

However, Sheet-metalNet and the incremental learning strategy proposed in this paper still have certain limitations:

(i) Although Sheet-metalNet outperforms hierarchical CADNet on the MFCAD++ dataset, achieving recognition accuracy of less than 99% suggests there is room for improvement, especially in the recognition accuracy of intersecting features. Future work will focus on addressing this shortcoming.
(ii) This research proposes using deep learning for implicit decisions in Automatic Feature Recognition (AFR). Future work aims to integrate fuzzy logic-based decision-making, given its robustness and capacity of dealing with uncertainty^52^, to potentially enhance AFR system performance.
(iii) The incremental learning strategy was intended for real industrial part datasets. However, this paper only verified its necessity and feasibility using synthetic datasets. In future work, collaborations with manufacturing companies will be made to build real industrial part datasets, improve related incremental learning experiments, and refine the incremental learning strategy for better performance.
(iv) There is still untapped potential in maFEG and GNNs. Future work will focus on exploring their applications in CAD model automatic generation and intelligent CAD model retrieval.

## Data Availability

The open-source dataset MFCAD++ dataset cited in this study is available from^53^. The SMCAD pretraining dataset created by this study, is available at https://doi.org/10.5281/zenodo.10976313. The SMCAD incremental dataset created by this study, is available at https://doi.org/10.5281/zenodo.10976392.

## Code availability

The code and specific details of all the experiments in this study are available at https://github.com/MaFangchang/deep-learning-based-MFR. The synthesis code for the SMCAD dataset is available at https://github.com/MaFangchang/SMCAD-dataset.
